# Non‐small cell lung cancer with mesenchymal–epithelial transition gene exon 14 skipping mutation treated with crizotinib

**DOI:** 10.1002/rcr2.453

**Published:** 2019-07-05

**Authors:** Seigo Katakura, Nobuaki Kobayashi, Kohei Somekawa, Nami Masumoto, Makoto Kudo, Takeshi Kaneko

**Affiliations:** ^1^ Department of Pulmonology Graduate School of Medicine, Yokohama City University Yokohama Japan; ^2^ Department of Pulmonology Yokohama City University Medical Center Yokohama Japan

**Keywords:** Crizotinib, MET exon 14 alterations, next‐generation sequence, NSCLC

## Abstract

We report the case of an 85‐year‐old man who was surgically diagnosed with lung adenocarcinoma (pT2aN1M0 stage IIA). He was administered platinum combination chemotherapy as first‐line treatment for lung cancer recurrence. The patient's pleural fluid sample was obtained and analysed using a next‐generation sequencer, which demonstrated the presence of mesenchymal–epithelial transition gene (MET) exon 14 skipping mutations. As the patient developed progressive disease after receiving first‐line chemotherapy, crizotinib was administered as the second‐line treatment. The treatment was effective, and the patient had a stable disease for 7 months. This case suggests that crizotinib is effective against non‐small cell lung cancer with MET exon 14 alterations.

## Introduction

Recently, various molecular targeted therapies have been developed and have shown positive effects against non‐small cell lung cancer (NSCLC) with a driver oncogene. Crizotinib, a multi‐targeted tyrosine kinase inhibitor, has been approved by regulatory agencies as a treatment for advanced NSCLC with anaplastic lymphoma kinase (ALK) or ROS1 rearrangements. Although crizotinib is not yet approved for use in NSCLC patients with mesenchymal–epithelial transition gene (MET) mutations, it was originally developed as a MET inhibitor. Hence, we report the case of a patient who had lung adenocarcinoma recurrence with a MET exon 14 (METex14) skipping mutation treated with crizotinib as a secondary chemotherapy drug. In this case, the administration of crizotinib was sufficient to control disease progression for 7 months.

## Case Report

**Figure 1:**
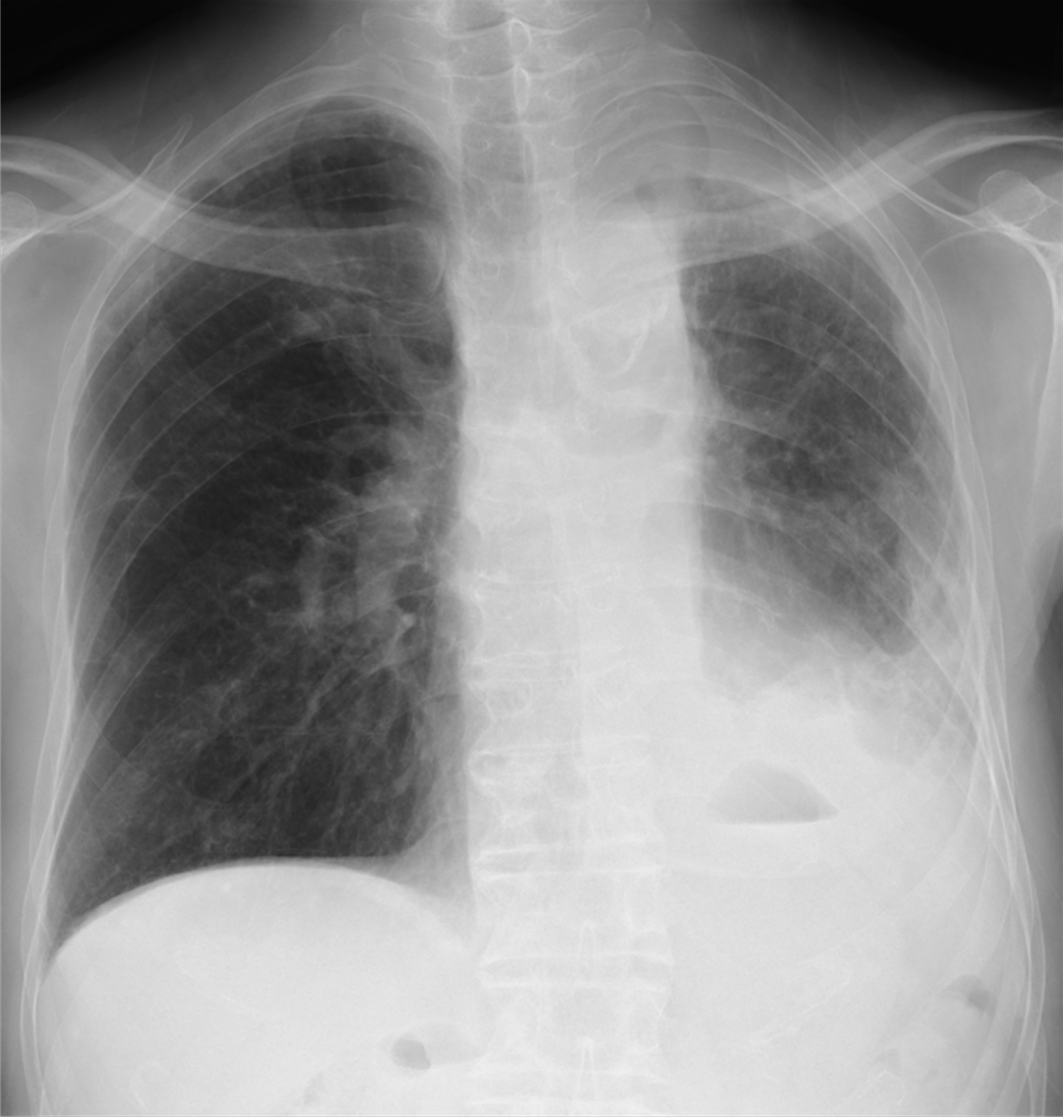
Chest X‐ray. Chest X‐ray showing a dull left costophrenic angle and decrease in left lung volume.

The patient was a non‐smoking 85‐year‐old Japanese man. He underwent lobectomy of the left upper lung and was diagnosed with lung adenocarcinoma (pT2N1M0 stage IIA) in October 2014. His lung cancer recurred in the left lower lobe, second lumbar bone, and left pleura in June 2016. A pleural fluid specimen was obtained and examined for the presence of genetic alterations using a next‐generation sequencer (NGS) and identified METex14 skipping mutations. Although the patient received four cycles of combination chemotherapy with carboplatin and pemetrexed (PEM), followed by one cycle of PEM as maintenance chemotherapy, in addition to pleurodesis with talc for malignant pleural effusion, computed tomography (CT) scan demonstrated progressive disease (PD) due to an increase in the number of multiple nodules in the lower left lobe and a left‐sided pleural effusion (Fig. 1). A CT scan also showed the presence of multiple nodules just below the left lower lobe and multiple bone metastases. Moreover, the patient had abnormal laboratory findings (haemoglobin: 9.2 g/dL, total protein: 6.8 g/dL, albumin: 3.9 g/dL, alkaline phosphatase: 322 U/L, blood urea nitrogen: 25 mg/dL, creatinine: 1.4 mg/dL, C‐reactive protein: 0.25 mg/dL, carcinoembryonic antigen: 13.5 ng/mL, and sialyl Lewis X: 120 U/mL).

**Figure 2:**
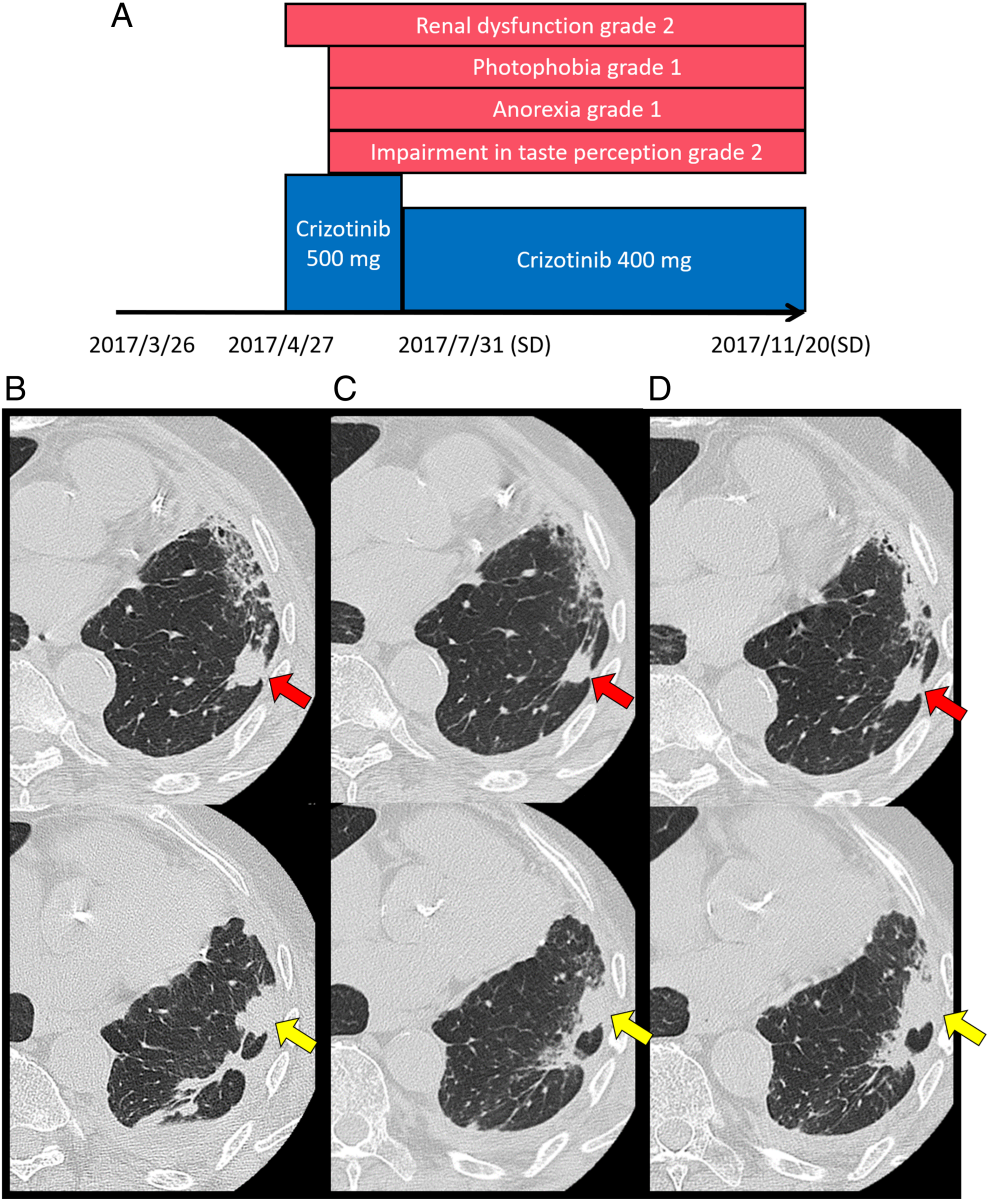
Clinical course after administration of crizotinib. (A) Clinical course and adverse events were presented after administration of crizotinib. (B) CT scan of the chest showing nodular lesions in the left lower lobe (red arrow, 15 × 15 mm; yellow arrow, 20 × 15 mm) after the initiation of crizotinib on 26 March 2017. (C) Both target lesions (red arrow, 12 × 11 mm; yellow arrow, 16 × 13 mm) decreased slightly on 31 July 2017. (D) Both target lesions (red arrow, 14 × 13 mm; yellow arrow, 20 × 15 mm) increased slightly on 20 November 2017.

He was excluded from the clinical trials administering MET inhibitors because of a coexisting stable prostate cancer. As regulatory agencies did not approve the use of crizotinib in NSCLC with MET mutations, crizotinib (500 mg/day) was administered as secondary chemotherapy without health insurance coverage after receiving the patient's consent on 27 April 2017 and after obtaining approval from the ethics committee of our hospital. On 4 May, the patient experienced impairment in taste perception (grade 2), anorexia (grade 2), and photophobia (grade 1). However, these adverse events eventually resolved without any specific treatments. The dosage of crizotinib was reduced from 500 to 400 mg/day on 25 May due to the occurrence of renal dysfunction (almost grade 3). On 31 July, the target lesion was slightly reduced as shown on the patient's CT scan, and the patient's disease remained stable. On 20 November, the target lesion was slightly increased in size as shown on the patient's CT scan, but there was no evidence of disease progression. Although his disease was controlled with crizotinib for 7 months, the treatment was discontinued as the patient developed anorexia and experienced financial difficulties (Fig. 2). Because the patient did not recover, he could not receive tertiary chemotherapy, and he therefore received best supportive care. He was transferred to the palliative care unit on 31 May 2018 and died on 16 June. Overall survival from the initiation of crizotinib was 13.5 months.

## Discussion

MET is an oncogene that encodes a receptor tyrosine kinase using a hepatocyte growth factor (HGF) as a ligand. The activation of HGF/MET signalling pathway is involved in the survival, proliferation, migration, and invasion of cancer cells. METex14 alterations are very diverse. These alterations can be caused by loss, insertion, or replacement of the nucleotide sequence at the splicing site of MET exon 14 RNA and lead to increased MET protein stability and oncogenic potential 1, 2. According to a previous study evaluating the comprehensive genomic profiling of 11, 205 patients with NSCLC in the United States, NSCLC with METex14 alterations was identified in 297 patients (2.7%), and approximately 70% of them had adenocarcinoma 3. Crizotinib has already been approved for use in patients with advanced NSCLC with ALK or ROS1 rearrangements but not for patients with NSCLC with METex14 alterations. In contrast, crizotinib was considered a MET inhibitor as it was initially developed to inhibit MET phosphorylation in vitro and in vivo 4. Hence, the present study focused on evaluating the inhibitory effects of crizotinib against MET. On May 2018, Pfizer Inc. announced that the United States Food and Drug Administration granted breakthrough therapy designation to crizotinib for the treatment of metastatic NSCLC patients with METex14 alterations that were experienced on or after platinum‐based chemotherapy.

Several studies reported the efficacy of crizotinib in NSCLC patients with METex14 alterations (Table 1). In this review, NSCLC patients with METex14 alterations who received crizotinib had an overall response rate of 71% (27 of 38 patients) 2, 3, 5, 6, 7, 8, 9, 10, 11, 12.

**Table 1:** Characteristics of NSCLC patients with METex14 alterations treated with crizotinib in the previous reports.

Author	Histology	METex14 alteration	Best response	Reference
Paik et al.	Adeno	3024_3028del	PD	2
Paik et al.	Adeno	V1001_F1007del	PR	2
Paik et al.	Adeno	3028G>T	PR	2
Schrock et al.	Adeno	3028+1_3028+delG	PR	3
Schrock et al.	Adeno	D1010Y	PR	3
Schrock et al.	Adeno	3028+1delG	CR	3
Schrock et al.	Adeno	D1010H	SD	3
Schrock et al.	Adeno	2888‐16_2888‐3del14	PR	3
Schrock et al.	Squamous	2888‐11_2904del28	PR	3
Schrock et al.	Adeno	2888‐16_2888‐13delTTCT	CR	3
Schrock et al.	Adeno	3028+1G>A	PR	3
Tan et al.	Adeno	3025–3028+5del	PR	5
Lu et al.	Adeno	D1228N, Y1230H, Y1230S, G1163R	PR	6
Jenkins et al.	Adeno	2887‐18_2887‐7del12	PR	7
Jorge et al.	Adeno	1010+1G>A	PR	8
Waqar et al.	Adeno	D1028H	PR	9
Mahjoubi et al.	Adeno	D1028N	PR	10
Awad et al.	Adeno	3028G>A	PR	11
Liu et al.	Sarcomatoid	Intron 14+3 A>G	PR	12

CR, complete response; METex14, MET exon 14; NSCLC, non‐small cell lung cancer; PD, progressive disease; PR, partial response; SD, stable disease.

Here, we report the case of a Japanese NSCLC patient with METex14 alterations who was successfully treated with crizotinib. This Asian elderly patient had stable disease for at least 7 months and developed minor adverse events after treatment with crizotinib alone. This case suggests that crizotinib is also effective in this patient population as reported in previous studies. After the ongoing phase II multicentre clinical trial in Japan, crizotinib might be approved for use in patients with advanced NSCLC with METex14 alterations.

### Disclosure Statement

Appropriate written informed consent was obtained for publication of this case report and accompanying images.
